# Association between Dietary Inflammatory Index and serum Klotho concentration among adults in the United States

**DOI:** 10.1186/s12877-022-03228-8

**Published:** 2022-06-27

**Authors:** Chichen Zhang, Zilong Zhang, Jiakun Li, Linghui Deng, Jiwen Geng, Kun Jin, Xiaonan Zheng, Shi Qiu, BiRong Dong

**Affiliations:** 1 Department of Urology, Institute of Urology and National Clinical Research Center for Geriatrics, West China Hospital of Sichuan University, No. 37, Guoxue Lane, Chengdu, 610041 Sichuan Province People’s Republic of China; 2 National Clinical Research Center of Geriatrics, The Center of Gerontology and Geriatrics, West China Hospital, Sichuan University, Chengdu, 610041 Sichuan People’s Republic of China; 3 Department of Gerontology, West China Hospital of Sichuan University, Chengdu, 610041 Sichuan Province People’s Republic of China; 4 Department of Nephrology, and National Clinical Research Center for Geriatrics, West China Hospital of Sichuan University, Guoxue Alley, No. 37, Chengdu, 610041 Sichuan People’s Republic of China

**Keywords:** Klotho, Dietary intake, Inflammation, Biomarker of aging, NHANES

## Abstract

**Background:**

Klotho is a hormone that emerges as an antiaging biomarker. However, the influence of the dietary pattern’s inflammatory potential on serum Klotho levels in human populations, especially in a general adult population, remains unknown. This study aimed to evaluate the relationship between the dietary inflammatory index (DII) and serum Klotho concentrations in individuals living in the United States.

**Methods:**

From the 2007–2016 National Health and Nutrition Examination Survey database, data of participants who completed the full 24-h dietary history and underwent serum Klotho testing were analyzed. The association between DII and serum Klotho concentrations was estimated using multivariable linear regression models. We also conducted segmented regression model to examine the threshold effect of DII on serum Klotho concentrations.

**Results:**

A total of 10,928 participants were included, with a median serum Klotho concentration of 805.20 pg/mL (IQR: 657.58 − 1001.12) and a median DII of 1.43 (IQR: − 0.16 − 2.82). Multivariable regression showed that participants with high DII scores were associated with low serum Klotho concentrations; when classifying DII into quartiles, after full adjustment, participants in DII quartiles 3 and 4 showed a decrease in Klotho levels (25.27 and 12.44 pg/ml, respectively) compared with those in the lowest quartile (quartile 1) (95% CI: − 41.80, − 8.73 and − 29.83, 4.95, respectively; *P* for trend = 0.036). The segmented regression showed that the turning point value of DII was − 1.82 (95% CI: − 2.32, − 0.80). A 1-unit increase in DII was significantly associated with lower Klotho levels by − 33.05 (95% CI: − 52.84, − 13.27; *P* = 0.001) when DII ranges from − 5.18 to − 1.82; however, the relationship was not significant when DII ranges from − 1.82 to 5.42 (*P* > 0.05). Furthermore, stratified analyses indicated that the observed associations between DII and serum Klotho concentration were stronger among those aged ≥ 56 years, those with normal weight, and those without chronic kidney disease (*P* for interaction = 0.003, 0.015, and 0.041, respectively).

**Conclusions:**

In summary, we indicated that there was a dose–response relationship between DII and serum Klotho concentrations, suggesting that adhering to an anti-inflammatory diet has beneficial effects on aging and health by increasing the serum Klotho concentration.

**Supplementary Information:**

The online version contains supplementary material available at 10.1186/s12877-022-03228-8.

## Highlights

1. klotho deficiency linked to multiple premature-aging syndromes and a short lifespan
2. Proinflammatory cytokines, including TNF-α, IFN-γ, and IL-6, downregulate α-Klotho gene expression
3. Dietary Inflammatory Index was developed as a tool to determine the inflammatory potential of diet
4. We found that there was a dose–response relationship between DII and circulating concentrations of klotho in a nationally representative population of American adults.

**Supplementary Information:**

The online version contains supplementary material available at 10.1186/s12877-022-03228-8.

## Introduction

Alpha-Klotho (Klotho) is a single-pass transmembrane protein encoded by the *klotho* gene. It is highly expressed in the distal convoluted tubules of the kidney and acts as an obligate coreceptor for fibroblast growth factor 23 (FGF23) [1, 2]. Kuro-o et al. first identified Klotho as a regulator of senescence in a mouse model with *k*lotho gene expression defects resulting in multiple premature-aging syndromes and a short lifespan [1]. Conversely, the *k*lotho gene also suppress aging in mammals, and its overexpression is related to an extended lifespan of up to 30% in transgenic mice [3]. Klotho has been emerged as an antiaging biomarker and functions as a hormone; it also paticipates in suppressing oxidative stress and inflammation [4]. Klotho deficiency is linked to numerous aging-related diseases, such as cancers, osteoporosis, cardiovascular and renal diseases, and neurodegenerative disorders [5–7]. Recently, low serum Klotho concentration was found to be associated with all-cause mortality in a nationally representative sample of American adults [8].

Currently, dietary patterns play an important role in life expectancy and reduce the risk of overall mortality and morbidity [9, 10]. Proinflammatory cytokine secretion, especially tumor necrosis factor-α (TNF-α), interferon γ (IFN-γ), and interleukin-6 (IL-6) [11], downregulates *k*lotho gene expression [12]. Through histone acetylation, inflammatory cytokines could cause the epigenetic inactivation of Klotho transcription [13]. Diets high in fruits, vegetables, nuts, and whole grains are associated with decreased inflammation [14]. In a previous study, nut consumption was positively associated with serum Klotho levels [15]. Thus, a proinflammatory diet may reduce the level of serum Klotho, which is used as an indicator of *k*lotho gene expression [4]; such reduction may accelerate aging. The Dietary Inflammatory Index (DII) is a recent developed standardized scoring system used to evaluate tools characterizing the inflammatory potential of a person’s diet by using a quantitative scale from anti- to proinflammation [16]. It was validated in diverse populations to predict the levels of inflammatory markers such as C-reactive protein, TNF-α, and IL-6 [17–19]. In addition, DII was associated with a wide range of other inflammatory-related diseases, such as obesity, various cancers, cardiovascular diseases, and shortened telomere length [20–23]. However, studies that investigated the association between the DII and serum Klotho levels are scarce, and the results are inconsistent. Jurado-Fasoli et al. found that DII was positively associated with serum Klotho in middle-aged adults [24] but negatively associated in young sedentary healthy adults [25].

Therefore, the insight into whether the inflammatory potential of the dietary pattern could influence serum Klotho levels in human populations, especially in a general adult population, remains unknown. Therefore, this study aimed to evaluate the association between dietary inflammatory potential and serum Klotho concentrations in a nationally representative sample of adults in the United States. We hypothesized that individuals with a high proinflammatory diet would have a lower Klotho level than those with a high anti-inflammatory diet.

## Material and method

### Sample population

The National Health and Nutrition Examination Survey (NHANES) is a major ongoing program of the National Center for Health Statistics (NCHS) at the Centers for Disease Control and Prevention. NHANES is designed to assess the health and nutritional status of noninstitutionalized residents in the United States. It uses a complex, stratified, multistage, probability sampling design to identify participants representative of the target population. It is unique in such a way that it not only collects questionnaire data through in-person interviews but also performs health examinations in a mobile examination center and collects specimens for laboratory tests. More details about NHANES can be found on their website (https://www.cdc.gov/nchs/nhanes/about_nhanes.htm). This study obtained written informed consent in all participants and was approved by the NCHS Ethics Review Board.

In this study, we limited individual data to five continuous NHANES cycles: 2007–2008, 2009–2010, 2011–2012, 2013–2014, and 2015–2016 (50,588 in total). Serum klotho was only measured among those aged 40–79 years who consented to surplus serum collection for future research. Thus, participants who completed the full 24-h dietary history and underwent serum Klotho testing were included. We excluded those with incomplete data on dietary recall assessments (*n* = 6,600) and serum klotho (*n* = 31,027). We also excluded participants who were diagnosed with cancer or stroke. Ultimately, 10,928 participants were included in the final analyses (Supplementary Fig. 1).

### Assessment of dietary inflammatory index

Dietary intake information was collected in NHANES using 24-h dietary recall interviews conducted at the Mobile Examination Center. The Food Surveys Research Group of the US Department of Agriculture is responsible for the methodology of dietary data collection, maintenance of the databases used to code and process the data, and data review and processing. Shivappa N et al. reported the development and validation of the DII [16, 18]. DII scores were generated using the first 24-h dietary information was used to generate DII scores [16]. In this study, 27 of 45 food parameters were available through the NHANES database, including protein; fat; alcohol; carbohydrates; fiber; cholesterol; omega-3 and omega-6 polyunsaturated fatty acids; saturated, monounsaturated, and polyunsaturated fatty acids; niacin; vitamins A, B1, B2, B6, B12, C, D, and E; iron; magnesium; zinc; selenium; folic acid; beta carotene; and caffeine. Each parameter labeled with an inflammatory effect score and the methodology of calculating DII are described in detail in Supplementary Material. The DII scores’ calculation was based on the intake of each food component as expressed per 1000 cal food consumed, also known as the energy-adjusted dietary inflammatory index (E-DII) [26]. In total, the DII score reflects the properties of the participant’s dietary intake from anti-inflammation (low DII scores) to proinflammation (high DII scores) [16].

### Serum Klotho concentration

Serum specimens were stored at -80 °C at the Centers for Disease Control and Prevention in Atlanta, GA. They were shipped on dry ice to the Northwest Lipid Metabolism and Diabetes Research Laboratories at the University of Washington in Seattle, WA, during the period 2019–2020. Klotho concentrations were determined on frozen NHANES 2007–2016 samples that were tested through a commercially available ELISA kit produced by IBL International, Japan [27]. All samples were analyzed twice, and the final value was calculated using the average of the two concentrations. Two quality control samples, including low and high Klotho concentrations, were also analyzed twice in each plate. The analysis results were automatically transmitted from the instrument to the laboratory Oracle Management System for the area supervisor to evaluate. Samples with duplicate values exceeding 10% were flagged to be retested. If the value of a quality control sample was not within two standard deviations (SDs) of the assigned value, the sample analysis was repeated. The assay sensitivity was 6 pg/mL, and all the final values of the samples exceeded this limitation, so no imputation was performed.

### Study covariates

Potential confounders were selected a priori according to a literature review [23, 28, 29]. Sociodemographic characteristics such as age, sex, self-reported race/ethnicity (Mexican American, Other Hispanic, non-Hispanic White, non-Hispanic Black, or other race), family income/poverty ratio, body mass index (BMI), physical activity, smoking status, alcohol intake, and estimated glomerular filtration rate (eGFR) were assessed. BMI was calculated according to self-reported weight and height measurements and was categorized into < 25, 25–29.9, or ≥ 30 kg/m^2^. Physical activity was classified into less than moderate (0 min/week moderate-to-vigorous physical activity [MVPA]), moderate active (< 150 min/week MVPA), and vigorous active (≥ 150 min/week MVPA) [30]. In accordance with the NCHS classifications, individuals were categorized into never, former, and current smokers [31]. Alcohol intake was categorized as none (0 g/d), moderate (male: 0.1 to 27.9 g/d, female: 0.1 to 13.9 g/d), and heavy drinking (male: ≥ 28 g/d, female: ≥ 14 g/d) [31]. Furthermore, the participants’ kidney function was evaluated by measuring the eGFR using the new Chronic Kidney Disease Epidemiology Collaboration Eq. [32]. The urinary albumin/creatinine ratio (ACR) was also calculated. According to the extended definition of chronic kidney disease (CKD), participants with an ACR ≥ 30 mg/g or an eGFR ≤ 60 ml/min/1.73m^2^ had CKD [28].

### Statistical analyses

We used the appropriate survey procedures to account for the complex sampling design and weights in NHANES (https://wwwn.cdc.gov/nchs/nhanes/analyticguidelines.aspx). Continuous variables are presented as the mean ± SD or median and interquartile range (IQR) when appropriate, whereas categorical variables are expressed as percentages. The differences between the DII quartile groups were compared using the Student’s-t test or Mann–Whitney U test for the continuous variables and the chi-square or Fisher’s exact test for the categorical variables.

The association between DII and serum Klotho concentrations was estimated using multivariable linear regression models including the nonadjusted model (model I), minimally adjusted model II (only age, sex, and race were adjusted), and fully adjusted model III (age, sex, race, family income/poverty ratio, BMI, alcohol intake, physical activity, smoking status, and eGFR were adjusted). DII was treated as both a continuous independent variable and a categorical variable (divided into quartiles), with the lowest quartile used as the reference. Linear trend tests were performed by entering the median value of each DII category as a continuous variable in the models. We further applied smooth curve fitting and segmented regression (also known as piece-wise regression) model to examine the threshold effect of DII on serum Klotho concentrations. Log-likelihood ratio test that compares one-line (non-segmented) model with segmented regression model was also conducted. To explore whether the association was modified by age, sex, BMI, physical activity, and CKD, we conducted interaction analyses and stratified analyses. Moreover, the effects of outliers (serum Klotho and DII) on our results were assessed by sensitivity analysis. We tested the stability of our results after excluding those with Klotho levels > 2500 pg/ml, those with Klotho levels > 2000 pg/ml, and those with Klotho levels > 2000 pg/ml and DII > 5.

All statistical data were analyzed using the software packages R (http://www.R-project.org, The R Foundation) and Empower (R) (www.empowerstats.com; X&Y Solutions, Inc., Boston, MA). A two-tailed *P* value < 0.05 was considered statistically significant.

## Results

Table 1 shows the population characteristics and sorted by DII quartiles. Overall, 10,928 participants were included, with a median age of 56.00 (47.00–65.00) years, and 48.54% were males. The median DII was 1.43 (–0.16– 2.82), ranging from –5.18 to 5.42. The median serum Klotho concentration was 805.20 (657.58–1001.12) pg/ml. The median eGFR was 90.80 (76.29–102.37) ml/min/1.73m^2^, and 18.26% of the participants had CKD. Serum Klotho concentrations were similar between the DII quartile groups. Participants in the highest DII group were more likely to be older, female, non-Hispanic black, and obese, have never smoked, have a lower eGFR, have a PIR < 1.3, and have less than moderate physical activity than those in the lowest DII group (all *P* < 0.05).

**Table 1:** Characteristics of participants in the 2007–2016 NHANES

Characteristic	Total	Quartile 1	Quartile 2	Quartile 3	Quartile 4	*P* value
Number	10,928	2732	2732	2732	2732	
DII (median, IQR)	1.43 (-0.16–2.82)	-1.24 (-2.06–0.67)	0.69 (0.28–1.08)	2.13 (1.79–2.48)	3.57 (3.17–4.08)	< 0.001
Age (median, IQR), years	56.00 (47.00–65.00)	55.00 (47.00–63.00)	55.00 (47.00–64.00)	56.00 (47.00–65.00)	57.00 (48.00–66.00)	< 0.001
Klotho (median, IQR), pg/ml	805.20 (657.58–1001.12)	815.30 (666.98–1002.60)	803.25 (655.65–1006.35)	802.85 (654.15–984.85)	799.20 (651.58–1009.03)	0.245
eGFR (median, IQR) mL/min/1.73m^2^	90.80 (76.29–102.37)	91.85 (77.83–102.52)	91.59 (77.53–102.67)	90.04 (75.25–102.03)	89.61 (74.72–102.42)	< 0.001
**% Sex**						< 0.001
male	48.55%	61.79%	53.22%	44.88%	34.30%	
female	51.45%	38.21%	46.78%	55.12%	65.70%	
**% Race**						< 0.001
Mexican American	17.16%	18.70%	18.23%	17.09%	14.60%	
Other Hispanic	12.20%	11.27%	11.79%	12.81%	12.92%	
Non-Hispanic White	41.16%	41.84%	42.39%	40.15%	40.26%	
Non-Hispanic Black	20.19%	16.00%	18.16%	21.23%	25.37%	
Other Race	9.30%	12.19%	9.44%	8.71%	6.84%	
**% PIR**						< 0.001
< 1.3	30.03%	23.38%	26.44%	32.00%	38.31%	
1.3–3.5	35.98%	31.79%	35.96%	37.51%	38.67%	
> 3.5	33.99%	44.83%	37.59%	30.49%	23.01%	
**% BMI**						< 0.001
normal (< 25 kg/m^2^)	23.42%	26.31%	23.58%)	21.73%	22.07%	
overweight (25–29.9 kg/m^2^)	35.28%	37.10%	36.25%)	35.14%	32.62%	
obesity (> = 30 kg/m^2^)	41.30%	36.59%	40.17%)	43.13%	45.31%	
**% Physical activity**						< 0.001
less than moderate	56.26%	43.95%	55.11%	60.50%	65.45%	
moderate	8.77%	9.50%	8.79%	8.87%	7.91%	
vigorous	34.98%	46.55%	36.09%	30.63%	26.65%	
**% Smoking status**						< 0.001
Never	52.68%	54.51%	53.11%	51.59%	51.50%	
Former	31.05%	34.10%	32.50%	30.72%	26.89%	
Current	16.27%	11.39%	14.39%	17.69%	21.61%	
**% Alcohol intake (gm)**						< 0.001
none	76.21%	68.19%	72.40%	78.11%	86.13%	
moderate	8.06%	10.98%	9.00%	6.55%	5.71%	
heavy	15.73%	20.83%	18.59%	15.34%	8.16%	
**CKD**^a^						< 0.001
No	81.74%	86.01%	82.92%	81.21%	76.76%	
Yes	18.26%	13.99%	17.08%	18.79%	23.24%	

Median (IQR) for continuous variables: *P* value for Mann–Whitney U test

% for Categorical variables: *P* value for chi-square test

DII quartile ranges: Quartile 1 = -5.18 to -0.16; Quartile 2 = -0.16 to 1.43; Quartile 3 = 1.43 to 2.82; Quartile 4: 2.82 to 5.42

*DII* Dietary Inflammatory Index, *PIR* Ratio of family income to poverty, *BMI* Body Mass Index

^a^chronic kidney disease (CKD) was defined as albumin-to-creatinine ratio (ACR) above 30 mg/g or estimated glomerular filtration rate (eGFR) below 60 mL/min/1.73 m.^2^

Table 2 presents the associations between DII and serum Klotho concentration. Participants with a higher DII score were associated with a lower serum Klotho concentration. After minimal and full adjustment, a 1-unit increase in DII was significantly associated with lower Klotho levels by 4.52 and 4.39 pg/ml in models 2 and 3, respectively (model 2: 95% CI: − 7.39, − 1.65, *P* = 0.002; model 3: 95% CI: − 7.49, − 1.28, *P* = 0.006). Moreover, the significant association persisted when classifying DII into quartiles. After adjustment for age, sex, and race, the DII was negatively associated with serum Klotho concentration (*P* for trend = 0.015). Compared with those at quartile 1, those at quartiles 2 and 3 had significantly decreased klotho levels (15.17 and 32.62 pg/ml) (95% CI: − 30.29, − 0.04; *P* = 0.049; 95% CI: − 48.30, − 16.95; *P* < 0.001, respectively). In the fully adjusted model, participants in the highest DII quartile had a 12.44 pg/ml decrease in Klotho levels compared with those in the lowest quartile (*P* for trend = 0.036; 95% CI: − 29.83, 4.95; *P* = 0.161). Additionally, the β values for serum Klotho concentration among participants in DII quartiles 2 and 3 were 9.75 and 25.27 pg/ml, respectively, compared with those in the lowest quartile. (95% CI, − 25.53, 6.03, *P* = 0.226; 95% CI, − 41.80, − 8.73, *P* = 0.003, respectively). A nonlinear relationship between DII and serum Klotho concentrations was observed by smoothing curve fitting after fully adjustment. (Supplementary Fig. 2) The segmented regression showed that the turning point value of DII was − 1.82 (95% CI: − 2.32, − 0.80). A 1-unit increase in DII was significantly associated with lower Klotho levels by − 33.05 (95% CI: − 52.84, − 13.27; *P* = 0.001) when DII ranges from − 5.18 to − 1.82; however, the relationship was not significant when DII ranges from − 1.82 to 5.42 (*P* > 0.05). (Log likelihood ratio test: 0.004; Table 3) In sensitivity analysis, the significant association persisted after excluding participants with Klotho levels > 2500 pg/ml, those with Klotho levels > 2000 pg/ml, and those with Klotho levels > 2000 pg/ml and DII > 5, respectively (data not shown).

**Table 2:** Association between Dietary Inflammatory Index and serum Klotho concentrations among adults in NHANES 2007–2016

**Dietary Inflammatory index group**	Model 1		Model 2		Model 3	
	β(95%CI)	*P*-value	β(95%CI)	*P*-value	β(95%CI)	*P*-value
Continuous	-1.59 (-4.40, 1.22)	0.268	-4.52 (-7.39, -1.65)	0.002	-4.39 (-7.49, -1.28)	0.006
Quartiles						
Q1	0		0	0	0	0
Q2	-9.39 (-24.58, 5.80)	0.226	-15.17 (-30.29, -0.04)	0.049	-9.75 (-25.53, 6.03)	0.226
Q3	-22.53 (-38.14, -6.92)	0.005	-32.62 (-48.30, -16.95)	< 0.001	-25.27 (-41.80, -8.73)	0.003
Q4	3.11 (-12.77, 19.00)	0.701	-12.41 (-28.59, 3.77)	0.133	-12.44 (-29.83, 4.95)	0.161
P for trend		0.626		0.015		0.036

95%CI: 95% Confidence interval

Model 1: no adjust

Model 2: adjusted for age; sex; Race

Model 3: adjusted for age; sex; Race; Ratio of family income to poverty; BMI; alcohol intake; physical activity; smoking; eGFR

DII quartile ranges: Quartile 1 = -5.18 to -0.16; Quartile 2 = -0.16 to 1.43; Quartile 3 = 1.43 to 2.82; Quartile 4: 2.82 to 5.42

**Table 3:** Threshold effect analysis for the relationship between Dietary Inflammatory Index and serum Klotho concentrations among adults in NHANES 2007–2016^a^

Models	serum Klotho concentrations	
	β(95%CI)	*P*-value
Model I		
One line slope	-4.63 (-7.61, -1.65)	0.002
Model II		
Turning point (K)	-1.82 (-2.32, -0.80)	
<—1.82 slope 1	-33.05 (-52.84, -13.27)	0.001
>—1.82 slope 2	-2.19 (-5.61, 1.24)	0.211
Slope 2 – Slope 1	30.87 (9.63, 52.11)	0.004
Predicted at 2.82	846.43 (834.62, 858.24)	
Log likelihood ratio test	0.004^b^	

Model I, linear analysis

Model II, non-linear analysis

Log likelihood ratio test: *P*-value < 0.05 means Model II is significantly different from Model I, which indicates a non-linear relationship

^a^adjusted for age; sex; Race; Ratio of family income to poverty; BMI; alcohol intake; physical activity; smoking; eGFR

^b^indicates that Model II is significantly different from Model I

In stratified analyses, the observed associations between DII and serum Klotho concentration were stronger among those aged ≥ 56 years, normal weight, and without CKD. (*P* for interaction = 0.003, 0.015, and 0.041, respectively) (Table 4). When we limited the analysis to individuals aged ≥ 56 years, participants within DII quartile 2, 3, and 4 were associated with a 30.10, 45.72, and 32.21 pg/ml Klotho level decrease when compared with DII quartile 1 (95% CI: − 49.18, − 11.02; − 65.20, − 26.24; − 52.61, − 11.81, respectively; *P* for trend < 0.001). A 1-unit increase in the DII was also associated with lower serum Klotho concentrations among individuals with normal weight (*P* for trend = 0.015), and the β was − 25.79 (95% CI: − 53.91, 2.32), − 60.43 (95% CI: − 90.66, − 30.21), − 49.89(95% CI: − 81.59, − 18.20) for quartiles 2, 3, and 4, respectively. Similar results were observed in individuals without CKD (*P* for trend = 0.002, *P* for interaction = 0.041). Those in DII quartiles 3 and 4 had a 31.21 and 16.99 pg/ml decrease in serum Klotho level, respectively, compared with those in quartile 1. (95% CI: − 46.95, − 15.47; 95% CI: − 33.78, − 0.21, respectively). However, the association between DII and serum klotho was not significantly modified by sex or BMI.

**Table 4:** Stratified analyses of association between Dietary Inflammatory Index and serum klotho concentrations (pg/ml) in NHANES 2007–2016^a^

Variable	Dietary Inflammatory index group, β (95%CI), Quartiles					P for interaction
	Quartile 1	Quartile 2	Quartile 3	Quartile 4	P for trend	
**Age (year)**^c^						0.003
40–56	0	19.12 (-0.48, 38.73)	-3.88 (-24.88, 17.12)	-0.02 (-22.28, 22.24)	0.667	
56–80	0	-30.10 (-49.18, -11.02)	-45.72 (-65.20, -26.24)	-32.21 (-52.61, -11.81)	< 0.001	
**Sex**						0.094
Male	0	-1.89 (-18.68, 14.90)	-33.26 (-51.94, -14.59)	-34.18 (-55.38, -12.98)	< 0.001	
Female	0	0.27 (-22.11, 22.65)	-9.46 (-31.78, 12.87)	-0.13 (-22.56, 22.30)	0.808	
**BMI (kg/m**^**2**^**)**						0.015
normal (< 25 kg/m^2^)	0	-25.79 (-53.91, 2.32)	-60.43 (-90.66, -30.21)	-49.89 (-81.59, -18.20)	< 0.001	
overweight (25–29.9 kg/m^2^)	0	-4.42 (-27.02, 18.18)	-18.79 (-42.72, 5.14)	-6.37 (-32.15, 19.41)	0.346	
obesity (> = 30 kg/m^2^)	0	15.76 (-6.21, 37.73)	1.03 (-21.50, 23.55)	2.37 (-21.07, 25.81)	0.901	
**Physical activity**						0.09
Less than moderate	0	-13.04 (-32.90, 6.83)	-23.77 (-43.81, -3.72)	-23.83 (-44.40, -3.26)	0.014	
Moderate	0	-30.33 (-77.01, 16.34)	-64.32 (-115.35, -13.29)	18.67 (-33.41, 70.75)	0.901	
Vigorous	0	15.11 (-6.26, 36.48)	-12.36 (-35.75, 11.03)	-14.68 (-40.48, 11.12)	0.187	
**CKD**^b^	0					0.041
No	0	-0.74 (-15.61, 14.13)	-31.21 (-46.95, -15.47)	-16.99 (-33.78, -0.21)	0.002	
Yes	0	-29.05 (-65.83, 7.73)	3.42 (-33.85, 40.68)	-12.34 (-49.27, 24.58)	0.923	

95%CI: 95% Confidence interval

DII quartile ranges: Quartile 1 = -5.18 to -0.16; Quartile 2 = -0.16 to 1.43; Quartile 3 = 1.43 to 2.82; Quartile 4: 2.82 to 5.42

^a^adjusted for age; sex; Race; Ratio of family income to poverty; BMI; alcohol intake; physical activity; smoking status; eGFR. In each stratification, the model is not adjusted for itself

^b^chronic kidney disease (CKD) was defined as albumin-to-creatinine ratio (ACR) above 30 mg/g or estimated glomerular filtration rate (eGFR) below 60 mL/min/1.73 m.^2^

^c^the cutoff value was set by median

## Discussion

This study demonstrated there was a dose–response relationship between DII and serum Klotho concentrations; higher DII score was associated with a decreased concentration of serum Klotho on the left side of the turning point; however, the relationship was not significant on the right side of the turning point. Furthermore, stratified analyses showed that the observed association of DII and serum Klotho concentration was stronger and significantly differed by age, BMI, and CKD status.

Klotho is a transmembrane protein with important antiaging functions and anti-inflammatory effects [1, 13]. Recently, Kresovich et al. demonstrated that a lower circulating level of Klotho was associated with all-cause mortality, and the association was stronger in participants with lesser physical activities [8]. This study is the first to use NHANES data and include a general population sample of American adults. This study supported earlier evidence that a low serum level of Klotho is a marker of increased mortality rates. Chronic inflammation plays a major role in decreasing serum Klotho levels [13, 33]. Therefore, an anti-inflammatory diet might increase the serum Klotho level; high Klotho levels may delay aging. Inconsistent with our results, Jurado-Fasoli et al. found a positive association between DII and serum Klotho according to a secondary analysis using the participants of the FIT-AGEING project. However, they only included 73 middle-aged healthy sedentary adults (40–65 years old), and they were unsure whether the study results can be extended to different populations (i.e., younger/older, unhealthy, or physical activity) [24]. In their further study, they observed a negative association between Mediterranean diet and serum Klotho level in sedentary middle-aged adults (45–65 years old) [15]; however, traditional Mediterranean diet is associated with a lower incidence of age-related diseases and higher levels of life expectancy [34]. Similar to our results, they found an association between high intake of nuts, an anti-inflammatory food, and high serum Klotho levels [15]. In addition, a proinflammatory dietary pattern was found negatively associated with serum Klotho levels in 139 young adults [25]. In Hsu et al.’ study, resveratrol, which is primarily found in grapes and red wine, induced renal Klotho expression both in vivo and in vitro [35].

In our study, DII was negatively associated with serum Klotho on the left side of the turning point in a general population sample. Aging was also a potential modifier that influenced this negative association. This association was stronger in the older group, possible because natural aging results in a decrease in nephron number, causing the serum Klotho level to decrease; Klotho is an important factor involved in the aging process [36]. In other words, older should pay more attention to dietary habit, reducing red meat and incorporating something green with every meal. Our study also suggested that associations may vary by BMI. Interestingly, the Klotho level appeared to be decreased even more among individuals with normal weight compared with those with overweight and obesity. One possible explanation is that fat tissue is the main source of proinflammatory mediators and that excessive visceral adipose tissue could lead to chronic inflammation, thereby decreasing the serum Klotho level [37]. Therefore, a proinflammatory diet might induce a significant reduction in serum Klotho levels in those with normal weight. However, unlike previous studies, our study found that physical activity did not modify the relationship between DII and serum Klotho concentrations. This relationship was stronger in individuals without CKD than in those with CKD. Klotho is secreted mainly in the distal convoluted tubules, and low Klotho levels are independently associated with kidney function decline [38]. Additionally, serum Klotho levels declined with CKD progression, starting as early as stage 2 CKD [39]. In CKD progression, increasing FGF23 expression and decreasing the active vitamin D levels suppress Klotho expression [40]. Our finding could be explained by DII leading to a more apparent decrease in Klotho concentration in normal participants. Furthermore, our findings are in line with the results of Shivappa et al., who used data from NHANES and reported that a proinflammatory diet was related to a shortened leukocyte telomere length, which is also a marker of biological aging and has been validated to increase all-cause mortality [23]. Currently, studies investigating diet potential and serum Klotho levels in human populations remain limited.

The possible mechanisms for our findings may be through the effect of diet on inflammatory markers. Recent research has identified that both systemic and local inflammations decrease Klotho expression in the kidneys [13]. Moreno et al. revealed that Klotho is negatively regulated by inflammation in cell culture and in vivo. They found that proinflammatory cytokines, such as TNF-α and TNF-like weak inducer of apoptosis (TWEAK) could downregulate Klotho expression through the canonical activation of the inflammatory transcription factor nuclear factor kappa B (NF-κB) and specifically, a RelA-dependent mechanism [33]. Furthermore, Thurston et al. found that in vitro, TNF could significantly inhibit *klotho* gene expression secondary to inflammatory bowel disease, which was further potentiated by IFN-γ; in addition, an anti-TNF-α antibody attenuated inflammation and reversed the repression of Klotho expression [41]. Klotho also acts as a coreceptor necessary for FGF-23 function within the kidney [42, 43]. Indeed, FGF23 is a potent negative regulator of Klotho expression. Marsell et al. conducted a microarray analysis of gene expression in the kidneys of FGF23-overexpressing transgenic mice and found that Klotho mRNA was the most significantly decreased transcript [44]. Proinflammatory cytokines, including IL-1, IL-6, and TNF-α, might induce FGF23 expression through NF-κB activation [11, 45, 46]. They could also induce hepcidin secretion in the liver. Abnormally high levels of hepcidin could cause functional iron deficiency. Iron is a negative regulator of FGF23 levels, and iron deficiency increases FGF23 production independent of inflammation, probably by increasing the expression and stability of hypoxia-inducible factor 1α (HIF1α), which binds to hypoxia-responsive elements in the promoter region of *Fgf23* and induces its transcription [47]. Therefore, a more proinflammatory diet may elevate the cumulative inflammatory burden and result in the downregulation of *klotho* gene, thereby decreasing the Klotho level, especially in people who were above 60 years, have less than moderate physical activity, and have no CKD.

This is the first to evaluate the dietary potential of inflammation in relation to serum Klotho concentration using a nationally representative US population. We conducted the threshold effect analysis, and a dose–response relationship was found between DII and serum Klotho concentrations. However, it also has some limitations. First, the cross-sectional nature precluded the establishment of causality. Second, the NHANES database only included participants aged 40–80 years old who were tested for Klotho level measurement; therefore, we do not know whether our results can be applied to younger age groups. Third, dietary information was only evaluated once at a 24-h period, not taking into account the variability of day-to-day diet. Fourth, the distribution of serum Klotho was not normalized. However, considering the small sample size of outliers, the results were stable after the sensitivity analysis, which we excluded participants with Klotho levels > 2500 pg/ml, those with Klotho level > 2000 pg/ml, and those with Klotho level > 2000 pg/ml and DII > 5. Finally, although we adjusted for as many confounders as possible, some unmeasured confounders were still found.

In conclusion, there was a dose–response relationship between DII and serum Klotho concentrations in a nationally representative population of American adults, suggesting that adhering to an anti-inflammatory diet has beneficial effects on aging and health by increasing the serum Klotho concentration. Future human research is warranted to determine the causal relationship between DII and serum Klotho concentration.

## Supplementary Information

**Additional file 1:** **Supplementary figure 1.** Participants selection flowchart.

**Additional file 2:** **Supplementary figure 2.** Association between Dietary Inflammatory index and serum klotho concentrations among adults in NHANES 2007-2016.

**Additional file 3:** **Supplementary Material.** The method of the calculation of Dietary Inflammatory Index.  

## Data Availability

The data sets generated and/or analyzed during the current study are available from the NHANES repository, https://www.cdc.gov/nchs/nhanes/.

## Abbreviations

Klotho: Alpha-Klotho
FGF23: Fibroblast growth factor 23
TNF-α: Tumor necrosis factor-α
IFN-γ: Interferon γ
IL-6: Interleukin-6
DII: Dietary Inflammatory Index
NHANES: National Health and Nutrition Examination Survey
BMI: Body mass index
eGFR: Glomerular filtration rate
MVPA: Moderate-to-vigorous physical activity
CKD: Chronic kidney disease
SD: Standard deviations
IQR: Interquartile range
NF-κB: Factor nuclear factor kappa B
