# Supplementary figures and images for: Association between Dietary Inflammatory Index and serum Klotho concentration among adults in the United States
Source: BMC Geriatr. 2022 Jun 27;22:528. doi: 10.1186/s12877-022-03228-8 (PMC9238083; doi:10.1186/s12877-022-03228-8)

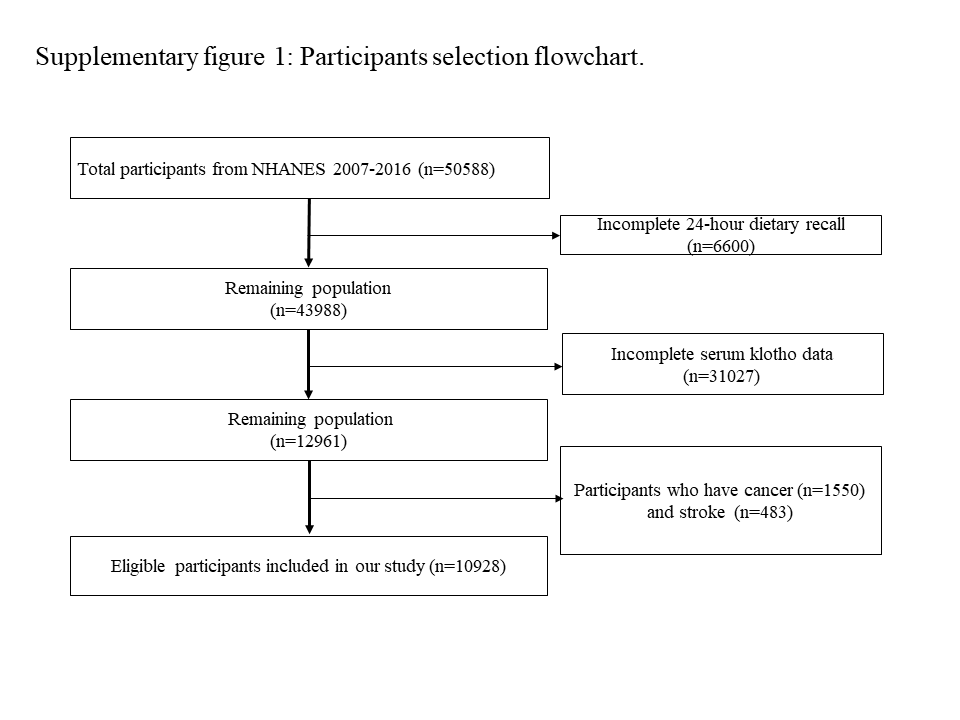

Supplement: Supplementary file 1 — Additional file 1: Supplementary figure 1. Participants selection flowchart. [file 12877_2022_3228_MOESM1_ESM.tif]

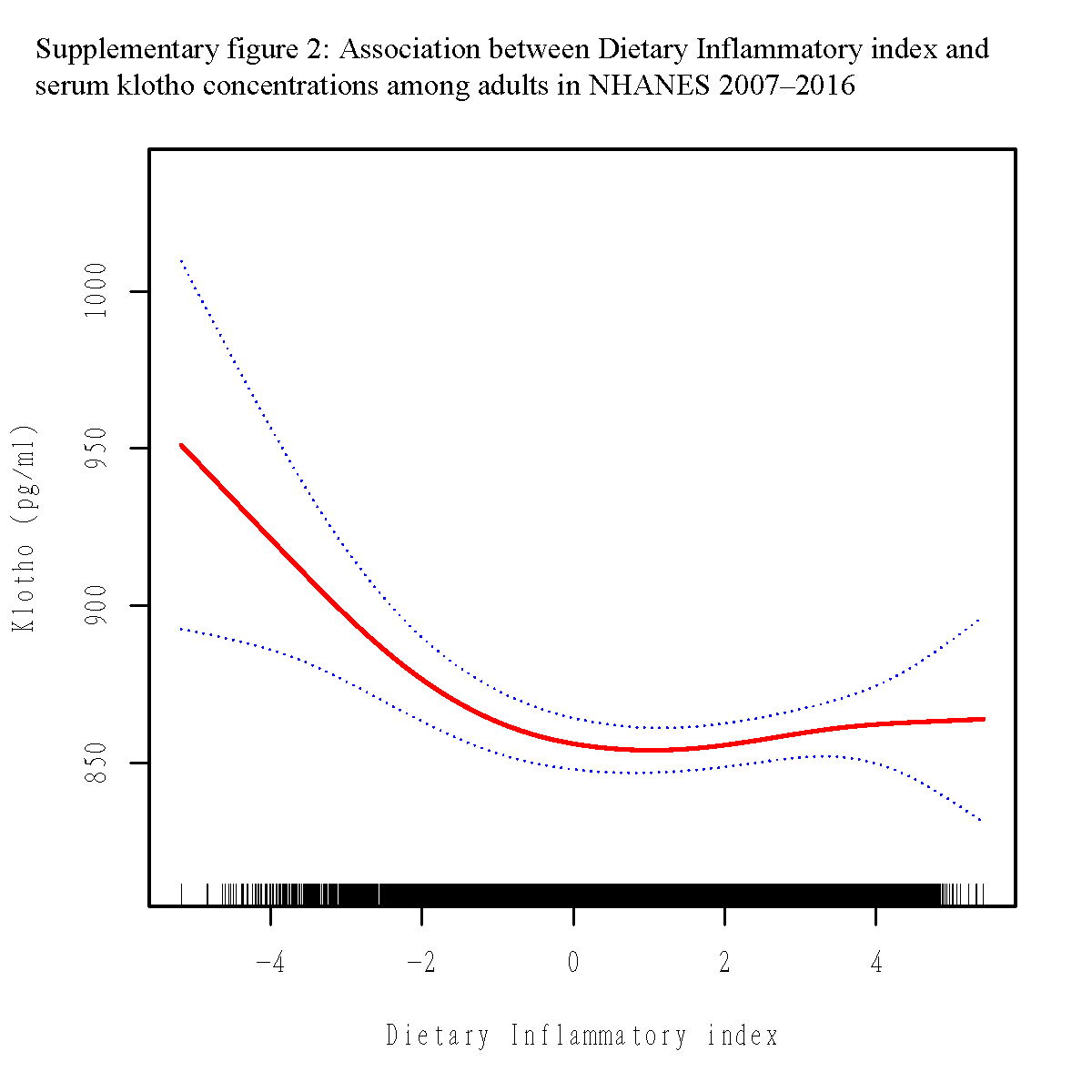

Supplement: Supplementary file 2 — Additional file 2: Supplementary figure 2. Association between Dietary Inflammatory index and serum klotho concentrations among adults in NHANES 2007-2016. [file 12877_2022_3228_MOESM2_ESM.tiff]
