# Supplementary material for: Association between Dietary Inflammatory Index and serum Klotho concentration among adults in the United States
Source: BMC Geriatr. 2022 Jun 27;22:528. doi: 10.1186/s12877-022-03228-8 (PMC9238083; doi:10.1186/s12877-022-03228-8)
Supplement: Supplementary file 3 — Additional file 3: Supplementary Material. The method of the calculation of Dietary Inflammatory Index. [file 12877_2022_3228_MOESM3_ESM.docx]

**The method of the calculation of Dietary Inflammatory Index**

Dietary Inflammatory Index (DII) calculation is standardized to a regionally representative world database which included daily dietary intake from 11 populations all over the world. Both standard mean and standard deviation were provided for all DII food parameters from the world database. For each of the 45 foods parameter, subtracting the individualized consumption value from the standard mean and dividing this value by the standard deviation generates a z-score. These Z scores were converted to proportions (with values from 0 to 1) to minimize the effects of positive skewing. To fulfill a symmetrical distribution centered around zero with bounds between -1 and +1, each proportion was doubled and then 1 was subtracted. Following, this value was multiplied by the corresponding inflammatory effect score for each food parameter.
